# Changes in cholesterol homeostasis modify the response of F1B hamsters to dietary very long chain n-3 and n-6 polyunsaturated fatty acids

**DOI:** 10.1186/1476-511X-10-186

**Published:** 2011-10-21

**Authors:** Jaime L Lecker, Nirupa R Matthan, Jeffrey T Billheimer, Daniel J Rader, Alice H Lichtenstein

**Affiliations:** 1 Cardiovascular Nutrition Laboratory, Jean Mayer USDA Human Nutrition Research Center on Aging at Tufts University, Boston MA, USA; 2 Cardiovascular Institute, University of Pennsylvania School of Medicine, Philadelphia PA, USA

## Abstract

**Background:**

The plasma lipoprotein response of F1B Golden-Syrian hamsters fed diets high in very long chain (VLC) n-3 polyunsaturated fatty acids (PUFA) is paradoxical to that observed in humans. This anomaly is attributed, in part, to low lipoprotein lipase activity and is dependent on cholesterol status. To further elucidate the mechanism(s) for these responses, hamsters were fed diets containing supplemental fish oil (VLC n-3 PUFA) or safflower oil (n-6 PUFA) (both 10% [w/w]) and either cholesterol-supplemented (0.1% cholesterol [w/w]) or cholesterol-depleted (0.01% cholesterol [w/w] and 10 days prior to killing fed 0.15% lovastatin+2% cholestyramine [w/w]).

**Results:**

Cholesterol-supplemented hamsters fed fish oil, relative to safflower oil, had higher non-high density lipoprotein (HDL) cholesterol and triglyceride concentrations (P < 0.001) which were associated with lower hepatic low density lipoprotein (LDL) receptor, sterol regulatory element binding protein (SREBP)-1c and acyl-CoA: cholesterol acyl transferase-2 (ACAT) mRNA and protein (p < 0.05), and higher hepatic apolipoprotein (apo) B-100 and apo E protein levels. In contrast, cholesterol-depleted hamsters fed fish oil, relative to safflower oil, had lower non-HDL cholesterol and triglyceride concentrations (P < 0.001) which were associated with lower hepatic SREBP-1c (p < 0.05) but not apo B-100, apo E or ACAT-2 mRNA or protein levels. Independent of cholesterol status, fish oil fed hamsters had lower HDL cholesterol concentrations (p < 0.001), which were associated with lower hepatic apoA-I protein levels (p < 0.05).

**Conclusion:**

These data suggest disturbing cholesterol homeostasis in F1B hamsters alters their response to dietary fatty acids, which is reflected in altered plasma lipoprotein patterns and regulation of genes associated with their metabolism.

## Background

The response of F1B hamsters to dietary very long chain n-3 polyunsaturated fatty acids (VLC n-3 PUFA), eicosapentaenoic acid (EPA) and docosahexaenoic acid (DHA), is dependent on cholesterol status and in some cases has been reported to be paradoxical to that observed in humans [1]. In humans with hypertriglceridemia, fish oil supplementation results in plasma triglyceride lowering and little change or a small increase in low density lipoprotein (LDL) cholesterol concentrations [2]. This effect is attributed to a reduction in the production rate of very low density lipoprotein (VLDL) [3]. In contrast, F1B hamsters fed diets high in cholesterol and VLC n-3 PUFA, relative to n-6 PUFA, dramatically increased triglyceride and non-HDL cholesterol concentrations [1,4-7]. This hypertriglyceridemic effect has been attributed, in part, to lower lipoprotein lipase activity which impedes triglyceride clearance rates [8]. Because the response to n-3 PUFA in hamsters affects both plasma triglyceride and cholesterol concentrations, it likely reflects events occurring in both the liver and small intestine. Of note, in the absence of supplemental dietary cholesterol, hamsters fed VLC n-3 PUFA had either comparable or more favorable plasma lipoprotein profiles relative to n-6 PUFA fed hamsters [1,4,5].

The regulation of plasma lipoprotein concentrations is a complex process. Hepatic cholesterol metabolism is tightly controlled by a balance between cholesterol synthesis, uptake and secretion, primarily involving the activities of 3-hydroxy-3-methyl-glutaryl (HMG)-CoA reductase, LDL receptor and 7α-hydroxylase, respectively [9,10]. Egress of hepatic triglyceride is mediated by microsomal triglyceride transfer protein (MTP) via the formation and secretion of nascent VLDL particles containing apolipoprotein (apo) B-100 and apo E [11-13]. Sufficient hepatic cholesterol is essential for VLDL formation [14,15]. Acyl-CoA cholesterol acyl transferase (ACAT)-2 generates hepatic cholesteryl ester [16]. Sterol regulatory element binding protein (SREBP)-1c and SREBP-2 regulate the expression of genes involved in hepatic fatty acid and cholesterol synthesis, respectively [17]. In cell culture systems and some animal models, PUFA inhibit the expression of SREBP-1 [18-20]. VLC n-3 PUFA appears to be more potent than n-6 PUFA, as suggested by lower expression of enzymes involved in the lipogenic pathway [18-21].

Intestinal cholesterol absorption also modulates plasma lipoprotein concentrations. The family of ATP-binding cassette (ABC) transporters, ABCA1, ABGG5 and ABCG8, regulate sterol absorption by facilitating the efflux of sterols from the apical (ABCG5/8) [22] or basolateral (ABCA1) [23] membrane of the enterocyte. Niemann-Pick C1 Like1 (NPC1L1) facilitates intestinal sterol uptake on the apical side of the enterocyte [24].

In both the liver and intestine, high-density lipoprotein (HDL) metabolism is mediated by ABCA1 and scavenger receptor class B type 1 (SR-B1) activities. ABCA1 enriches the cholesterol content of lipid-poor HDL particles by facilitating the efflux of hepatic and intestinal free cholesterol [25]. SR-B1 promotes the selective hepatic uptake of cholesteryl ester from HDL particles [26,27].

The aim of this work was to identify mechanisms associated with the differential response of the F1B hamster to dietary VLC n-3 and n-6 fatty acids as altered by dietary cholesterol. To address this aim we manipulated *in vivo *cholesterol homeostasis with the intent of stimulating (cholesterol depleted) or suppressing (cholesterol supplemented) hepatic cholesterol biosynthesis.

## Methods

### Animals and diets

Sixty-four 8 week-old male F1B Golden-Syrian hamsters (BioBreeders, Watertown, MA) were divided into four diet groups on the basis of body weight and housed in stainless steel suspended cages (4 hamsters/cage) with a reverse 12:12 light:dark cycle. Hamsters were given free access to LM-485 mouse/rat diet (Harlan-Teklad, Madison, WI) and water during a two-week acclimation period. Thereafter the hamsters were switched to *ad libitum *semi-purified diets containing 10% (w/w) safflower oil (n-6 PUFA) or low cholesterol fish oil (Arista Industries, Inc., Wilton, CT) (VLC n-3 PUFA), in combination with 0.1% (w/w) cholesterol or 0.01% (w/w) cholesterol for 12 weeks (see Additional File 1: Table S1 [diet composition] and Table S2 [dietary fatty acid profile]). The analytical data were consistent with the intended diet composition.

During the last ten days of the feeding period, 0.15% lovastatin (Merck & Co., Inc. Rahway, NJ) and 2% cholestyramine (Bristol-Myers Squibb Co., Princeton, NJ) were added to the 0.01% cholesterol diets. The combination of lovastatin and cholestyramine has previously been demonstrated necessary to lower plasma cholesterol concentrations in the hamster [28,29]. The 0.1% cholesterol diet and 0.01% cholesterol plus lipid-lowering drug diet were designed to supplement (+C) and deplete (-C), respectively, cholesterol metabolism in the animals to alter cholesterol biosynthesis (safflower +C, fish +C, safflower -C and fish -C). A portion of the data from the safflower oil fed hamsters has appeared previously to address an unrelated experimental question [30].

After 12 weeks of diet treatment the hamsters were fasted (16 hours) and killed by CO_2 _inhalation. Livers were removed and cleaned. A portion was immediately used for nuclear and membrane protein extraction and the remaining segments were frozen in liquid nitrogen and stored at -80°C. Small intestines were removed, flushed with PBS, and the jejunum was isolated, placed in RNAlater (Qiagen, Valencia, CA) and stored at -80°C. The animal protocol was approved by the Institutional Animal Care and Use Committee of the Jean Mayer Human Nutrition Research on Aging, Tufts University.

### Plasma lipid and lipoprotein analysis

Retro-orbital blood was collected into EDTA-coated tubes from fasted hamsters (16 hours) under isoflurane anesthesia at 0, 6 and 12 weeks. Plasma total cholesterol, HDL cholesterol and triglyceride concentrations were determined on a Cobas Mira automated analyzer using enzymatic reagents (Roche Diagnostics, Indianapolis, IN). Non-HDL cholesterol was calculated as the difference between total and HDL cholesterol. Four plasma pools per diet group were created by combining plasma from 4 animals per pool for fast protein liquid chromatography (FPLC) analysis using two Superose 6 columns (Amersham Biosciences, Piscataway, NJ) as previously described [31]. The total cholesterol concentration of the FPLC fractions was measured using enzymatic reagents (Wako, Richmond, VA).

### Liver lipid composition

Liver lipids were extracted [32], and total and free cholesterol, and triglyceride concentrations were determined using enzymatic reagents (Wako and Roche Diagnostics) [33]. Cholesteryl ester was calculated as the difference between total and free cholesterol. Delipidated liver tissue was digested in 1N NaOH for the determination of protein using the bicinchoninic acid (BCA) assay (Pierce Inc., Rockford, IL).

### Cholesterol content of experimental diets

Lipids were extracted from desiccated aliquots of diet [32], and total cholesterol was determined by gas chromatography (GC) as previously described [34].

### Fatty acid profiles

Fatty acid profiles of red blood cell membranes and experimental diets were determined as previously described [30].

### Quantitative real time PCR

Total RNA was extracted from the liver and jejunum using the Qiagen RNeasy Mini kit. A DNase digestion step was included to eliminate contamination with genomic DNA. RNA was reverse transcribed using SuperScript II reverse transcriptase with random hexamers (Invitrogen, Carlsbad, CA). Primers for ACAT-2, apoA-I, apoB-100, beta-actin, CYP7A1, HMG-CoA reductase, LDL receptor, MTP and SREBP-2 were designed using Primer Express software (Applied BioSystems, Foster City, CA), and primer specificity and amplification efficiency were verified before use. Real time PCR was conducted in an Applied Biosystems 7300 Sequence detection system using SYBR green reagents (Applied BioSystems) with the primers listed in Additional File 1: Table S3 [35,36]. Reaction conditions were 95°C for 10 minutes, 40 cycles of 95°C for 15 seconds and 60°C for 1 minute. A standard curve was included on all plates for each mRNA of interest and used to calculate relative levels. Values were normalized using beta-actin as an endogenous control.

### Immunoblotting analysis

Freshly excised liver tissue from 2 hamsters was pooled, and nuclear and membrane proteins and cell lysates were extracted as described previously [29,30]. Protein concentrations were determined using the BCA assay. Proteins were separated by SDS-PAGE and transferred to polyvinylidene difluoride membranes using a wet transfer system and detected as previously described [35]. Relative protein levels were normalized to the density of beta-actin.

### Statistical analysis

Data are expressed as means ± SEM. Prior to statistical analysis, data were checked for normality and appropriate transformations performed when necessary. Differences between dietary fat type (fish oil versus safflower oil) and cholesterol status (supplemented versus depleted) were determined using an unpaired Students t-test. Data that could not be normalized were analyzed using the Wilcoxon's signed rank test. Differences were considered significant at P ≤ 0.05. All statistical analyses were performed using SAS (Version 9.1, SAS Institute, Cary, NC).

## Results

### Plasma lipid and lipoprotein profiles

At baseline, plasma lipid and lipoprotein profiles were similar among the four hamsters groups (see Additional File 1: Table S4). The combination of lovastatin and cholestyramine resulted in 2- and 15-fold lower non-HDL cholesterol concentrations after drug treatment in safflower and fish oil fed hamsters, respectively (see Additional File 1: Tables S5).

**Figure 1:**
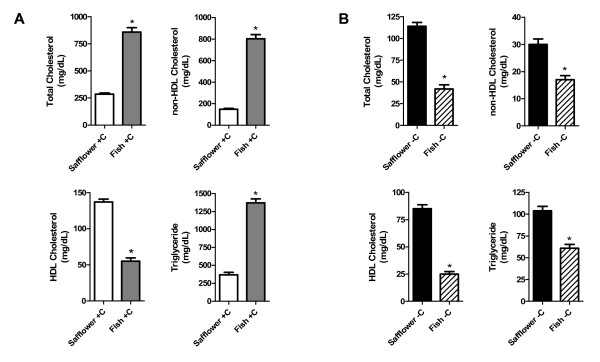
**Effect of dietary n-3 (fish oil) and n-6 PUFA (safflower oil) on fasting plasma lipid and lipoprotein cholesterol concentrations in cholesterol-supplemented (A) and cholesterol-depleted (B) hamsters**. Retro-orbital blood was collected into EDTA coated tubes from fasted hamsters. Plasma cholesterol and triglyceride concentrations were determined enzymatically. Bars represent means ± SEM, n = 15-16 animals per group. Appropriate transformations of the data (log HDL; square root total cholesterol, non-HDL cholesterol; inverse triglyceride) were made before statistical analysis. Asterisks indicate significant differences between safflower and fish oil within cholesterol-supplemented (+C) or depleted (-C) hamsters, P ≤ 0.05.

After 12 weeks of diet treatment, cholesterol-supplemented hamsters fed fish oil, relative to safflower oil, had significantly higher total cholesterol (3-fold), non-HDL cholesterol (3.7-fold) and triglyceride (5.4-fold), and lower HDL cholesterol (2.5-fold) concentrations (Figure 1A). In contrast, the cholesterol-depleted hamsters fed fish oil, relative to safflower oil, had significantly lower total cholesterol (2.7-fold), non-HDL cholesterol (1.7-fold) triglyceride (1.7-fold) and HDL cholesterol (3.4-fold) concentrations (Figure 1B).

**Figure 2:**
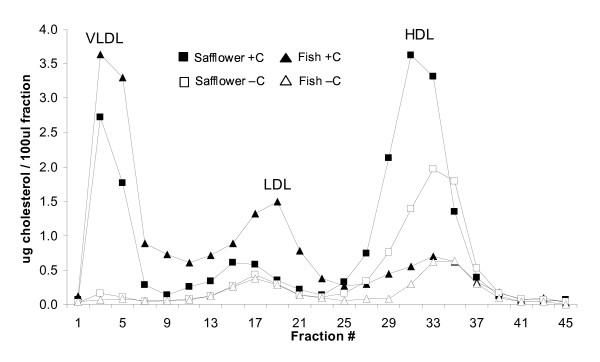
**Effect of dietary n-3 (fish oil) and n-6 PUFA (safflower oil) on fast protein liquid chromatography (FPLC) cholesterol profiles of plasma from cholesterol-supplemented (+C) or depleted (-C) hamsters**. Plasma (200 μL) was pooled from 4 hamsters and lipoprotein fractions were separated by FPLC. Cholesterol concentrations were measured in odd numbered fractions. Data represent the mean of 4-pooled groups.

Consistent with the plasma lipid and lipoprotein concentrations, FPLC patterns indicated that the cholesterol-supplemented hamsters fed fish oil, relative to safflower oil, carried more cholesterol in the VLDL and LDL fractions, and less in the HDL fraction (Figure 2). Conversely, the cholesterol-depleted hamsters fed fish oil, relative to the safflower oil, carried less cholesterol in all three lipoprotein fractions.

### Red blood cell fatty acid profile and hepatic lipid composition

Red blood cell membrane fatty acid profiles reflected that of the diet (Table 1). The mol% of n-3 and n-6 PUFA in the red blood cell membranes of hamsters fed fish oil were 17-18% and 14-15%, respectively, and of hamsters fed safflower oil were 1-2% and 38-39%, respectively. These data indicate that the diet treatment was of sufficient length to alter membrane composition.

**Table 1:** Red blood cell fatty acid profile^1^

Selected fatty acids	Safflower oil	Fish oil
**Total SFA^2^**	**mol% of total fatty acids**	
-C	41.9 ± 0.4	47.2 ± 0.4*
+C	39.1 ± 0.4	37.8 ± 0.6
16:0		
-C	26.5 ± 0.3	32.2 ± 0.4*
+C	25.2 ± 0.2	30.0 ± 0.3*
18:0		
-C	13.5 ± 0.1	12.5 ± 0.2*
+C	12.2 ± 0.2	8.6 ± 0.4*
Total MUFA^3^		
-C	17.9 ± 0.3	21.0 ± 0.3*
+C	20.3 ± 0.4	29.3 ± 0.5*
18:1		
-C	14.8 ± 0.2	17.3 ± 0.2*
+C	16.5 ± 0.2	24.0 ± 0.4*
Total n-6 PUFA^4^		
-C	37.6 ± 0.4	14.5 ± 0.2*
+C	38.5 ± 0.5	14.3 ± 0.2*
18:2n-6		
-C	16.6 ± 0.2	5.8 ± 0.2*
+C	19.6 ± 0.5	8.2 ± 0.2*
20:4n-6		
-C	15.9 ± 0.3	7.4 ± 0.1*
+C	14.0 ± 0.2	5.2 ± 0.3*
Total n-3 PUFA^5^		
-C	1.8 ± 0.1	17.0 ± 0.4*
+C	1.3 ± 0.1	18.5 ± 0.5*
20:5n-3		
-C	0.1 ± 0.03	5.9 ± 0.1*
+C	0.04 ± 0.01	8.8 ± 0.4*
22:6 n-3		
-C	1.4 ± 0.1	7.9 ± 0.2*
+C	0.9 ± 0.03	6.9 ± 0.1*

^1^Values are means ± SEM, n = 16 per group. Asterisks indicate significant differences between safflower oil and fish oil within cholesterol-supplemented (+C) or depleted (-C) hamsters, P ≤ 0.05. Data (Total MUFA, 20:4n-6, 20:5n-3) were log-transformed prior to statistical analysis.

^2^Sum of 8:0, 10:0, 12:0, 14:0, 16:0, 18:0, 20:0, 24:0

^3^Sum of 14:1n-5, 16:1n-9, 16:1n-7, 17:1n-7 18:1n-9, 18:1n-7, 24:1n-9

^4^Sum of 18:2n-6, 18:3n-6, 20:3n-6, 20:4n-6, 22:4n-6, 22:5n-6

^5^Sum of 18:3n-3, 20:5n-3, 22:5n-3, 22:6n-3

Cholesterol-supplemented hamsters fed fish oil, relative to safflower oil, had a 1.5-fold lower hepatic total and cholesteryl ester content (p < 0.05), and 1.8-fold higher hepatic triglyceride content (p < 0.05) (Table 2). In contrast, cholesterol-depleted hamsters fed fish oil, relative to safflower oil, had a 2-fold lower hepatic triglyceride content (p < 0.05), with small, but significant differences in hepatic cholesterol content. Combined with the differences observed in plasma lipoprotein concentrations these data suggest a disturbance in hepatic lipoprotein metabolism. Of note, dietary fat type had no significant effect on liver weights, suggesting no impairment in lipoprotein secretion.

**Table 2:** Liver lipid composition^1^

	Safflower oil	Fish oil
**Liver weight**	**Grams**	
-C	4.9 ± 0.1	4.8 ± 0.2
+C	7.1 ± 0.2	7.5 ± 0.3
Free cholesterol^2^	μg/mg protein	
-C	22 ± 0.5	19 ± 0.7*
+C	44 ± 3	44 ± 3
Cholesteryl ester		
-C	3 ± 0.3	6 ± 0.8*
+C	221 ± 24	149 ± 8*
Triglyceride		
-C	108 ± 7	52 ± 4*
+C	46 ± 3	83 ± 5*

^1^Values are means ± SEM, n = 14-16 per group. Asterisks indicate significant differences between safflower oil and fish oil within cholesterol-depleted (-C) or supplemented (+C) hamsters, P ≤ 0.05.

^2^Data were log-transformed prior to statistical analysis

### Hepatic and intestinal mRNA levels

**Figure 3:**
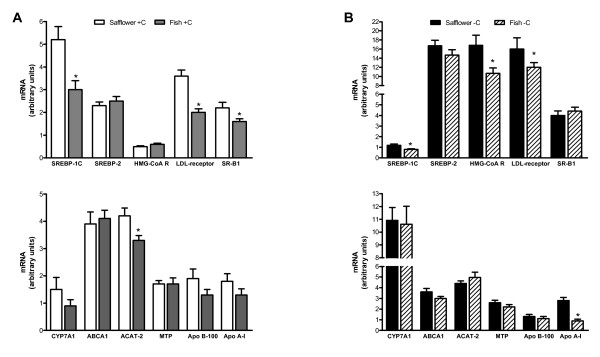
**Effect of dietary n-3 (fish oil) and n-6 PUFA (safflower oil) on hepatic mRNA levels of genes associated with cholesterol and lipoprotein metabolism in cholesterol-supplemented (A) and cholesterol-depleted (B) hamsters**. Real time PCR was used to measure gene expression in the liver. A standard curve was run on all plates for each mRNA of interest to calculate relative levels. Values were normalized using beta-actin as an endogenous control. Bars represent means ± SEM, n = 14-16 animals per group. Appropriate transformations of the data (log SREBP-2, CYP7A1, MTP, apo B-100, ABCA1, HMG-CoA reductase; square root SR-B1, SREBP-1c, apo A-I) were made before statistical analysis. Asterisks indicate significant differences between safflower oil and fish oil within cholesterol-supplemented (+C) or depleted (-C) hamsters, P ≤ 0.05.

Cholesterol-supplemented hamsters fed fish oil, relative to safflower oil, had modest but significantly lower hepatic mRNA levels of SREBP-1c (1.7-fold), LDL receptor (1.8-fold), SR-B1 (1.4-fold) and ACAT-2 (1.3-fold) (Figure 3A). Cholesterol-depleted hamsters fed fish oil, relative to safflower oil, had a modest but significantly lower hepatic mRNA levels of SREBP-1c (1.5-fold), apo A-I (3-fold) and HMG Co-A reductase (1.6-fold) (Figure 3B). No significant differences in hepatic apo B-100, MTP, SREBP-2, CYP7A1 or ABCA1 mRNA levels was observed between fish oil and safflower oil fed hamsters, regardless of cholesterol status. These data suggest that the altered plasma lipoprotein patterns observed in response to differences in dietary fatty acid were due in part to changes in regulation of the genes involved in cholesterol and lipoprotein synthesis, uptake and secretion.

### Hepatic protein levels

**Figure 4:**
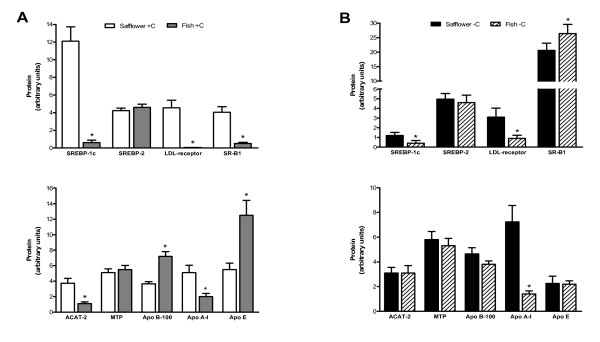
**Effect of dietary n-3 (fish oil) and n-6 PUFA (safflower oil) on hepatic protein levels of genes associated with cholesterol and lipoprotein metabolism in cholesterol-supplemented (A) and cholesterol-depleted (B) hamsters**. Proteins were extracted from the liver, separated by SDS-PAGE and detected by immunoblotting. Relative protein levels were normalized to the density of beta-actin. Bars represent means ± SEM, n = 14-16 animals per group. Appropriate transformations of the data (log apo A-I, apo E, LDL receptor, SREBP-1c, SR-B1; square root apo B-100) were made before statistical analysis. Asterisks indicate significant differences between safflower oil and fish oil within cholesterol-supplemented (+C) or depleted (-C) hamsters, P ≤ 0.05.

Cholesterol-supplemented hamsters fed fish oil, relative to safflower oil, had significantly lower hepatic protein levels of SR-B1 (8.2-fold), apo A1 (2.6-fold) and ACAT-2 (3.4-fold), lower hepatic membrane protein levels of SREBP-1c (20-fold) and LDL receptor (90-fold), and higher protein levels of apo B-100 (2-fold) and apo E (2.3-fold) (Figure 4A). Conversely, cholesterol-depleted hamsters fed fish oil, relative to safflower oil, had modest but significantly higher hepatic protein levels of SR-B1 (1.3-fold) (Figure 4B). Similar to the effect observed in cholesterol-supplemented hamsters, cholesterol-depleted hamsters fed fish oil, relative to safflower oil, had significantly lower hepatic apo A-I protein levels (5.1-fold), and lower membrane SREBP-1c (3-fold) and LDL receptor (3.4-fold) protein levels. Overall, differences in protein levels induced by dietary fat type were consistent with the changes observed in mRNA levels for the genes of interest.

**Figure 5:**
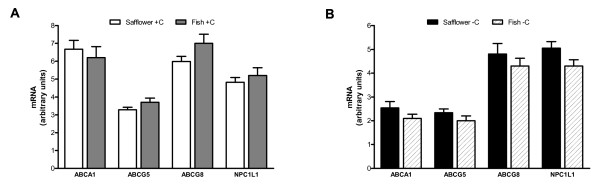
**Effect of dietary n-3 (fish oil) and n-6 PUFA (safflower oil) on mRNA levels of small intestinal sterol transporters in cholesterol-supplemented (A) and cholesterol-depleted (B) hamsters**. Real time PCR was used to measure gene expression in the small intestine. A standard curve was run on all plates for each mRNA of interest to calculate relative levels. Values were normalized using beta-actin as an endogenous control. Bars represent means ± SEM, n = 14-16 animals per group. Appropriate transformations of the data (log ABCG5) were made before statistical analysis. Asterisks indicate significant differences between safflower oil and fish oil within cholesterol supplemented (+C) or depleted (-C) hamsters, P ≤ 0.05.

In addition to hepatic gene expression, mRNA levels of intestinal sterol transporters were quantified to determine whether the differences attributable to dietary fat type and cholesterol status were contributed to by genes involved in cholesterol absorption. No significant differences in mRNA levels of ABCA1, ABCG5, ABCG8 and Niemann-Pick C1 Like1 (NPC1L1) were observed (Figure 5A, B).

## Discussion

F1B hamsters fed diets containing fish oil become severely hyperlipidemic [5], exacerbated when the diet also contains cholesterol [1,4,8]. This response has been attributed, in part, to decreased lipoprotein lipase activity and mRNA levels [8]. Our aim was to further identify the mechanisms for these observations. The focus was on expression of genes regulating cholesterol and lipoprotein metabolism, and intestinal cholesterol absorption. We took advantage of a model previously developed of disrupted cholesterol homeostasis to addresses the issues of interest [29,30].

Cholesterol-supplemented hamsters responded to diets containing fish oil, relative to safflower oil, with higher plasma non-HDL cholesterol concentrations. This observation was associated with lower hepatic LDL receptor mRNA and membrane protein levels, consistent with elevated non-HDL particle concentrations, as previously reported [5,36]. We further observed that although SREBP-2 has a regulatory role in LDL receptor transcription [37,38], the effect of fish oil on LDL receptor message levels was unrelated to SREBP-2 mRNA or nuclear protein levels.

Cholesterol supplemented hamster diets containing fish oil, relative to the safflower oil, also had dramatically higher hepatic triglyceride levels and plasma triglyceride concentrations. Nonetheless, SREBP-1c mRNA and membrane protein levels were lower in these hamsters. VLC n-3 PUFA have been reported to down-regulate SREBP-1c in both cell culture and animal models, which, in turn, leads to a reduction in the expression of genes involved in the fatty acid synthetic pathway [19-21,39]. One potential cause for the discordance in plasma and hepatic triglyceride concentrations and SREBP-1c expression is lower rates of hepatic fatty acid oxidation [40]. Similar to the results in the current study, fish oil did not decrease plasma triglyceride concentrations in apo E deficient mice, despite a reduction in the triglyceride production rate [41]. This suggests that down regulation of SREBP-1c alone does not account for the lower plasma triglyceride concentrations in this animal model.

Secretion of apo B containing lipoprotein particles is a major determinant of plasma non-HDL cholesterol and triglyceride concentrations, as well as hepatic lipid levels [42]. The assembly of VLDL particles is dependent upon the MTP [13], and the availability of apo B, apo E, and cholesterol [12,43]. There was no significant effect of dietary fat type, regardless of cholesterol status, on MTP mRNA or protein levels. However, the cholesterol-supplemented hamsters fed fish oil, relative to safflower oil, had higher hepatic apo B-100 and apo E protein levels. Apo E expression in mice is positively associated with the rate of hepatic VLDL production and secretion [41]. These data suggest a role of apo E in modulating plasma non-HDL cholesterol and triglyceride concentrations [44]. We also observed lower hepatic ACAT-2 protein expression in fish oil, relative to safflower oil, fed hamsters, which in turn may have contributed to lower hepatic cholesteryl ester concentrations. Taken together these data suggest that despite lower hepatic cholesterol available for VLDL synthesis, higher hepatic triglyceride, apo B and apo E levels may have contributed to the higher plasma triglyceride and non-HDL cholesterol concentrations in fish oil fed hamsters.

In addition to hepatic cholesterol metabolism, intestinal cholesterol absorption is also a determinant of plasma non-HDL cholesterol concentrations [22]. Nonetheless, no significant differences in mRNA levels of the sterol transporters ABCA1, ABCG5, ABCG8 and NPC1L1 were observed in response to dietary fat type in cholesterol-supplemented or cholesterol-depleted hamsters. These data imply that this was not a major regulatory point of plasma cholesterol concentrations in this animal model. Hamsters fed diets containing fish oil without cholesterol have been reported to have lower mRNA levels of NPC1L1 relative to hamsters fed control diets or diets containing olive oil [45], suggesting that the effect of fish oil on NPC1L1 expression may be secondary to whole body cholesterol status.

In cholesterol-supplemented hamsters, diets containing fish oil resulted in lower HDL cholesterol concentrations than safflower oil. Plasma HDL cholesterol concentrations is regulated, in part, by apo A-I, the major structural protein of HDL [46]. Hepatic apo A-I protein levels were lower in cholesterol-supplemented hamsters fed fish oil, relative to the safflower oil. ABCA1 and SR-B1 also modulate HDL cholesterol concentrations through mediating the production and catabolism of HDL particles, respectively [26,27,47]. The lower SR-B1 mRNA and protein levels observed in fish oil, compared to safflower oil fed, cholesterol-supplemented hamsters is not consistent with the lower HDL cholesterol concentrations in this group. These data suggest that, in these animals, regulation of HDL cholesterol uptake is primarily at the level of SR-B1 receptor activity. Higher SR-B1 activity in response to fish oil has been observed in the rat [48]. No significant differences in ABCA1 mRNA levels were observed in the current study. Post-transcriptional regulation of ABCA1 may be altered in response to fish oil; however, we were unable to detect ABCA1 protein in the liver samples.

Cholesterol-depleted hamsters responded differently to dietary fat type than did cholesterol-supplemented hamsters. Cholesterol-depleted hamsters fed fish oil, relative to safflower oil, had lower plasma and hepatic triglyceride concentrations. This was associated with lower hepatic SREBP-1c mRNA and protein levels, consistent with the known role of SREBP-1c in regulating plasma triglyceride concentrations [19].

There was an unanticipated effect of dietary fat type in cholesterol-depleted hamsters. Despite lower LDL receptor protein levels, the hamsters fed diets containing fish oil had lower non-HDL cholesterol concentrations than hamsters fed safflower oil. The difference in non-HDL cholesterol concentrations between fish oil and safflower oil fed hamsters was not be accounted for by differences in expression of genes modulating hepatic cholesterol synthesis and uptake, VLDL assembly and secretion, or intestinal cholesterol absorption. Both lovastatin and VLC n-3 PUFA inhibit HMG-CoA reductase activity. These factors may have contributed to the lower plasma non-HDL cholesterol concentrations in cholesterol-depleted hamsters fed fish oil [49-51].

The dramatic shift in cholesterol status of the hamster did not alter the effect of dietary fish oil, relative to safflower oil, on HDL cholesterol concentrations. Cholesterol-depleted hamsters fed diets containing fish oil, relative to safflower oil, had lower HDL cholesterol concentrations and this was associated with higher hepatic mRNA and protein levels of SR-B1, and lower hepatic protein apo A-I levels. This observation is consistent with previous findings that SR-B1 and apo A-I are major determinants of plasma HDL cholesterol concentrations [26,52,53].

## Conclusion

In conclusion, our findings indicated that higher non-HDL cholesterol and triglyceride concentrations in cholesterol-supplemented hamsters fed fish oil, relative to safflower oil, is associated with lower hepatic LDL receptor expression and higher hepatic apo E and apo B expression. In cholesterol-depleted hamsters, the hypolipidemic effect of fish oil is partly attributed to lower SREBP-1c expression. The lower HDL cholesterol concentrations in hamsters fed fish oil, relative to safflower oil, is independent of cholesterol status and is associated with lower hepatic apo A-I protein levels. There appears to be no correlate between the effect of VLC n-3 PUFA metabolism in humans and F1B hamsters.

## Competing interests

The authors declare that they have no competing interests.

## Authors' contributions

JLL participated in study design, implemented the study, conducted the statistical analysis, participated in data interpretation, and wrote the first draft of the manuscript. AHL supervised the study, and participated in study design and coordination, statistical analysis and data interpretation and manuscript preparation. NRM participated in study design and implementation, statistical analysis and data interpretation and manuscript review. DJR and JTB supervised the FPLC analysis. All authors read and approved the final manuscript.

## Supplementary Material

Additional file 1 **Supplementary Data for Methods and Results**. Data Tables detailing the Composition and Fatty Acid Profile of the Experimental Diets, Primer Sequences for Real Time PCR and Baseline, 6 week and 12 week Fasting Lipoprotein and Lipid Profiles Click here for file
