# Supplementary material for: Changes in cholesterol homeostasis modify the response of F1B hamsters to dietary very long chain n-3 and n-6 polyunsaturated fatty acids
Source: Lipids Health Dis. 2011 Oct 21;10:186. doi: 10.1186/1476-511X-10-186 (PMC3217862; doi:10.1186/1476-511X-10-186)
Supplement: Additional file 1 — Supplementary Data for Methods and Results. Data Tables detailing the Composition and Fatty Acid Profile of the Experimental Diets, Primer Sequences for Real Time PCR and Baseline, 6 week and 12 week Fasting Lipoprotein and Lipid Profiles [file 1476-511X-10-186-S1.DOC]

**Table S1**: Composition of experimental diets^1^

| Ingredient | 0.01% (w/w) cholesterol diet | 0.1% (w/w) cholesterol diet |
| --- | --- | --- |
|  | g/kg diet | |
| Casein | 204 | 203 |
| L-Methionine | 4 | 4 |
| Maltodextrin | 102 | 102 |
| Cornstarch | 281 | 281 |
| Sucrose | 129 | 129 |
| Cellulose | 122 | 122 |
| Safflower or fish oil | 100 | 100 |
| Soybean oil | 20 | 20 |
| Mineral mix, AIN-93GMX | 28 | 28 |
| Vitamin mix, AIN-93VX | 8.0 | 8.0 |
| Choline bitartrate | 2.0 | 2.0 |
| Cholesterol | 0.1 | 1 |
| Tert-butylhydroquinone | 0.02 | 0.02 |

^1^Semi purified diets were prepared by Research Diets, New Brunswick, NJ.

^2^Lovastatin (0.15% w/w) and cholestyramine (2.0% w/w) were added to the 0.01% (w/w) cholesterol diet the last ten days of treatment.

**Table S2**: Fatty acid composition of the diets^1^

| Selected Fatty Acids | Safflower oil | | Fish oil |
| --- | --- | --- | --- |
|  | Weight percent of total fatty acids | | |
| Total SFA^2,4^ |  |  | |
| –C^3^ | 14.4 | 33.8 | |
| +C | 14.4 | 33.3 | |
| 16:0 |  |  | |
| –C | 8.5 | 19.4 | |
| +C | 8.6 | 19.1 | |
| 18:0 |  |  | |
| –C | 5.5 | 7.8 | |
| +C | 5.4 | 7.7 | |
| Total MUFA^5^ |  |  | |
| –C | 16.8 | 23.6 | |
| +C | 16.8 | 23.6 | |
| 18:1n-9 |  |  | |
| –C | 16.7 | 15.5 | |
| +C | 16.8 | 15.5 | |
| Total n-6 PUFA^6^ |  |  | |
| –C | 66.8 | 12.4 | |
| +C | 66.9 | 12.5 | |
| 18:2n-6 |  |  | |
| –C | 66.9 | 11.1 | |
| +C | 66.8 | 11.1 | |
| 20:4 n-6 |  |  | |
| –C | ND | 0.6 | |
| +C | ND | 0.9 | |
| Total n-3 PUFA^7^ |  |  | |
| –C | 1.9 | 30.2 | |
| +C | 1.9 | 30.6 | |
| 18:3n-3 |  |  | |
| –C | 1.1 | 1.9 | |
| +C | 1.1 | 2.0 | |
| 20:5n-3 |  |  | |
| –C | ND | 15.1 | |
| +C | ND | 15.6 | |
| 22:6n-3 |  |  | |
| –C | ND | 10.8 | |
| +C | ND | 10.4 | |

^1^Values are averages of duplicate measurements of diet samples.

^2^Data were log-transformed prior to statistical analysis.

^3^–C, 0.01% (w/w) cholesterol diets; +C, 0.1% (w/w) cholesterol diets

^4^Sum of 8:0, 10:0, 12:0, 14:0, 16:0, 18:0, 20:0, 24:0

^5^Sum of 14:1n-5, 16:1n-9, 16:1n-7, 17:1n-7 18:1n-9, 18:1n-7, 24:1n-9

^6^Sum of 18:2n-6, 18:3n-6, 20:3n-6, 20:4n-6, 22:4n-6, 22:5n-6

^7^Sum of 18:3n-3, 20:5n-3, 22:5n-3, 22:6n-3

**Table S3**: Primers for quantitative real time PCR

| Gene | Forward Primer | Reverse Primer | Accession number/ Reference |
| --- | --- | --- | --- |
| ABCA1^1^ | ATAGCAGGCTCCAACCCTGAC | GGTACTGAAGCATGTTTCGATGTT | (35) |
| ABCG5^1^ | TGATTGGCAGCTATAATTTTGGG | GTTGGGCTGCGATGGAAA | (35) |
| ABCG8^1^ | TGCTGGCCATCATAGGGAG | TCCTGATTTCATCTTGCCACC | (35) |
| NPC1L1^1^ | CCTGACCTTTATAGAACTCACCACAGA | GGGCCAAAATGCTCGTCAT | (35) |
| SREBP-1c^2^ | GCGGACGCAGTCTGGG | ATGAGCTGGAGCATGTCTTCAAA | (35) |
| SREBP-2^2^ | GCAAGGTGTTCCTGCATGAA | TGGTGTTCTGACTGGTACGCC | GU12330 |
| LDL receptor^2^ | GCAGTGTTTCTGTGGCTGACAC | GCCATGCACAGGGTCCA | M94387 |
| HMG-CoA reductase^2^ | GAGCTACATTTGTGCTTGGCG | TTCATTAGGCCGAGGCTCAC | L00173 |
| SR-B1^2^ | AAGCCTGCAGGTCTATGAAGC | AGAAACCTTCATTGGGTGGGTA | (36) |
| ACAT-2^3^ | GGTGGAATTATGTGGCCAAGA | CATGTTGGCAAAGACAGGGAC | NM_153728 |
| CYP7A1^2^ | CACTCTGCACCTTGAGGATGG | GGGTCTGGGTAGATTGCAGG | L04690 |
| MTP^2^ | ACATGCTGACCTTTGTGCGA | ACGGTCATAATTGTGGGCAAC | U14995 |
| Apo A-I^2^ | GGCGGGAGATGAACAAGGA | GGCGGTAAAGAGCCACTTCC | AF046919 |
| Apo B-100^2^ | TGATTATCTGAATGCATCTGACTGG | TCCTTGGCATTGGCTACTTGT | AF176576 |
| Beta actin^2^ | TGCTGTCCCTGTATGCCTCTG | AGGGAGAGCGTAGCCCTCAT | AJ312092 |

^1^ Mouse sequence

^2^ Hamster sequence

^3^ Rat sequence

**Table S4**: Baseline non-fasting body weights and fasting plasma lipid and lipoprotein profile in F1B hamsters^1^

|  | Safflower oil | Fish oil |
| --- | --- | --- |
| Body weight (g) |  |  |
| –C | 96 ± 1 | 96 ± 1 |
| +C | 96 ± 1 | 96 ± 1 |
| Total cholesterol (mg/dl) |  |  |
| –C | 92 ± 2 | 98 ± 3 |
| +C | 98 ± 3 | 90 ± 3 |
| Non-HDL cholesterol^2^ (mg/dl) |  |  |
| –C | 35 ± 1 | 31 ± 2 |
| +C | 36 ± 2 | 33 ± 2 |
| HDL cholesterol (mg/dl) |  |  |
| –C | 57 ± 2 | 60 ± 2 |
| +C | 63 ± 2 | 65 ± 2 |
| Triglyceride^2^ (mg/dl) |  |  |
| –C | 94 ± 5 | 94 ± 4 |
| +C | 95 ± 5 | 94 ± 3 |

^1^Values are means ± SEM, n=16 per group. No statistically significant differences were observed among the groups at P≤ 0.05.

^2^Appropriate transformations of the data (inverse non-HDL cholesterol; log triglyceride) were made before statistical analysis.

**Table S5**: 6-week and 12- week fasting plasma lipid and lipoprotein profile in F1B hamsters1

|  | Safflower oil | | Fish Oil | |
| --- | --- | --- | --- | --- |
|  | 6-week | 12-week | 6-week | 12-week |
| Total cholesterol (mg/dl) |  |  |  |  |
| –C | 150 ± 3 | 114 ± 4 | 285 ± 15 | 42 ± 5 |
| +C | 247 ± 7 | 286 ± 11 | 668 ± 62 | 859 ± 40 |
| Non-HDL cholesterol (mg/dl) |  |  |  |  |
| –C | 56 ± 3 | 30 ± 2 | 230 ± 14 | 15 ± 1 |
| +C | 129 ± 6 | 149 ± 8 | 621 ± 64 | 804 ± 39 |
| HDL cholesterol (mg/dl) |  |  |  |  |
| –C | 94 ± 2 | 85 ± 4 | 55 ± 1 | 25 ± 2 |
| +C | 118 ± 3 | 137 ±4 | 47 ± 3 | 55 ± 5 |
| Triglyceride (mg/dl) |  |  |  |  |
| –C | 167 ± 14 | 104 ± 5 | 462 ± 41 | 61 ± 5 |
| +C | 326 ± 28 | 369 ± 33 | 1082 ± 113 | 1369 ± 60 |

^1^Values are means ± SEM, n=16 per group.
